# Supplementary material for: Child mental health predictors among camp Tamil refugees: Utilizing linear and XGBOOST models
Source: PLoS One. 2024 Sep 16;19(9):e0303632. doi: 10.1371/journal.pone.0303632 (PMC11404815; doi:10.1371/journal.pone.0303632)
Supplement: S1 Text — (DOC) [file pone.0303632.s001.doc]

**S1 Appendix**

**XGBoost Method**

Boosting is an ensemble learning technique to build a strong model from several weak classifiers in series. Boosting algorithms reduce both bias and variance by taking a weighted average of multiple weak learners. This idea has gained popularity over the years (Chen and Guestrin, 2016). The eXtreme Gradient Boosting method has become popular recently, and is dominating applied machine learning because of its scalability (Wang et al., 2022). XGBoost directly optimizes the following supervised learning loss function and controls the complexity of the trees by adding a regularization term to the objective function (for details see Chen and Guestrin, 2016),


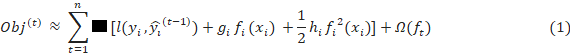


where
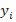
is the
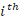
observed label,
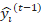
 is the
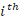
predicted label from the first t − 1 trees, *l* is the loss function,
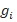
 is the first derivative of loss function,
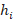
 is the second derivative of loss function,
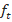
 is the tree output, and
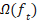
 is the regularization term defined as follows:


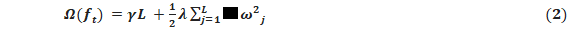


where
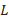
 is the number of leaf nodes,
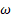
 is the leaf scores,
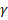
 and
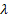
 are the hyperparameters. It is important to note that in the objective function of
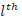
tree,
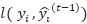
,
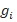
 and
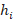
are constants.

**SHapley Additive exPlanations (SHAP) Method**

SHAP is a game theoretic approach used to explain the output of any machine learning model. It connects optimal credit allocation with local explanations using the classic Shapley values from game theory and their related extensions (Lundberg and Lee, 2017). SHAP creates simplified inputs by mapping
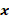
 to
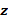
 through
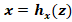
(Wang et al., 2022). Based on z, the original model
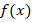
 can be approximated with a linear function of binary variables:


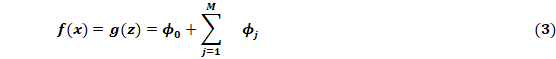


where
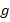
 is the explanation model,
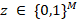
are the simplified features, M is the number of input features,
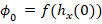
, and
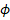
 is the feature attribution value for a feature j, the shapley value:


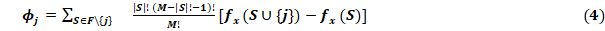


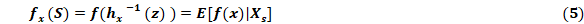


where F is the non-zero set of input in
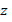
, S is the subset of
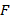
 (features used in the model) with
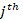
feature excluded from
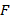
, and
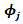
is the measure of additive feature attributions which is called the SHAP value. This method is widely used as it efficiently handles local accuracy, missingness and consistency. For more details on this method, see Lundberg et al., 2018.

Results from the linear analysis confirmed parental mental health and child total strengths and difficulties were significant predictors of child depressive symptoms. Additionally, results from XGBOOST suggested that, in addition to child total strengths and difficulties, family functioning variables were more important than parental depression in contributing to child depressive symptoms. There are several possible explanations for the partially inconsistent findings in the linear and XGBoost analysis. One reason may be that XGBoost is a non-linear boosting algorithm, so if it is showing an important predictor which is not significant in the linear model, that means, this particular variable is not linearly associated with the response, suggesting; the association of this particular variable with the response is much more complex.

We could conclude from our XGBoost results that all variables investigated contributed to the response, and they either have a (positively or negatively) high to moderate effect on the child’s depressive symptoms. Consistent with this result, the linear model analysis shows that two variables in the model (parental mental health and child total strengths and difficulties) most of these variables are significant in predicting child depressive symptoms with a moderate model performance. The model performance of the XGBoost model was also checked using the test data (20% of data) and the mean square error was less than 30%, which shows that the model did not perform poorly on the test data.


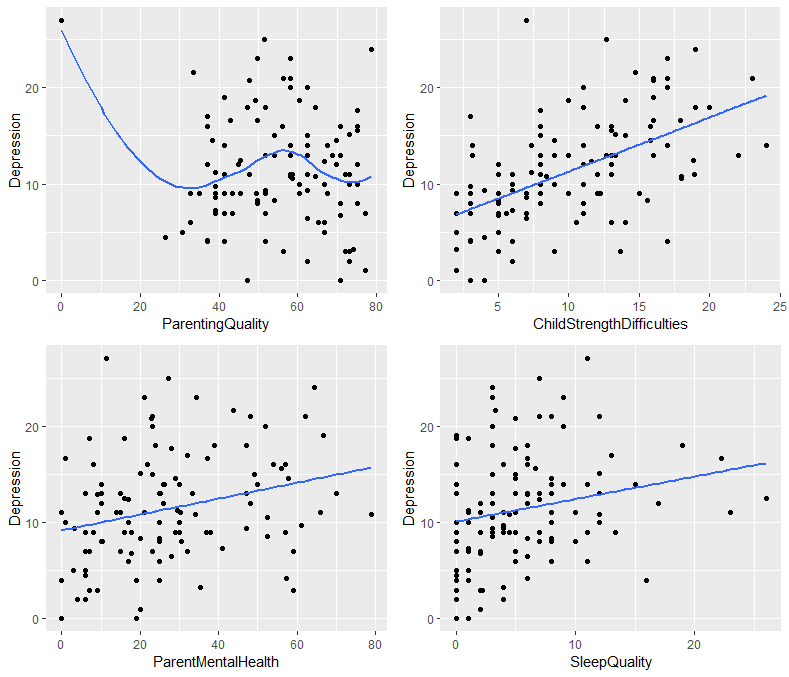


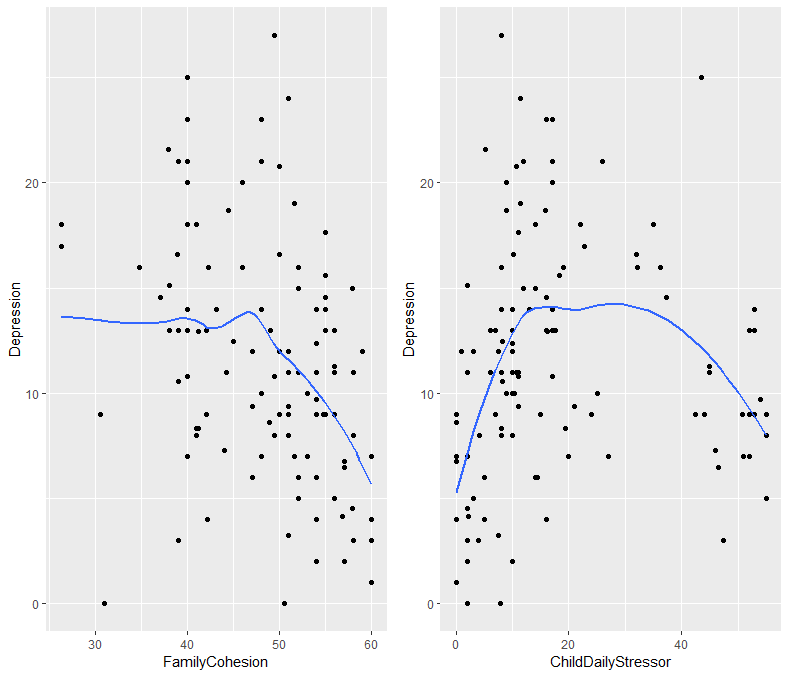


| Parenting quality | **-0.07984** |
| --- | --- |
| Parent mental health | 0.2692 |
| Child daily stressor | 0.04397 |
| Family cohesion | -**0.33496** |
