# Supplementary material for: Child mental health predictors among camp Tamil refugees: Utilizing linear and XGBOOST models
Source: PLoS One. 2024 Sep 16;19(9):e0303632. doi: 10.1371/journal.pone.0303632 (PMC11404815; doi:10.1371/journal.pone.0303632)
Supplement: Attachment — Submitted filename: Response to Reviewers-XGBoost models (2).docx [file pone.0303632.s003.docx]

Dear Editor,

Thank you for your response to our paper “*Child Mental Health Predictors Among Camp Refugees: Utilizing Linear and XGBOOST Models.”* Below is our response to the reviewers comments.

Journal Requirements:

When submitting your revision, we need you to address these additional requirements.

1. Please ensure that your manuscript meets PLOS ONE's style requirements, including those for file naming.

**Response: We have followed the format suggested. One reviewer asked us to deviate from the abstract format which we have ignored to stay with the suggested format requested by the editor.**

2. Note from Emily Chenette, Editor in Chief of PLOS ONE, and Iain Hrynaszkiewicz, Director of Open Research Solutions at PLOS: Did you know that depositing data in a repository is associated with up to a 25% citation advantage (<https://doi.org/10.1371/journal.pone.0230416>)? If you’ve not already done so, consider depositing your raw data in a repository to ensure your work is read, appreciated and cited by the largest possible audience. You’ll also earn an Accessible Data icon on your published paper if you deposit your data in any participating repository (<https://plos.org/open-science/open-data/#accessible-data>).

**Response: Thank you for this suggestion. We do plan on putting the data in a repository once we have completed the analysis of the data for future papers. We have multiple papers analyzing this data currently, and will make the data available once we have completed these manuscripts.**

3. Thank you for stating the following in the Acknowledgments Section of your manuscript:

"This work was supported by National institute of Health Fogarty International [grant numberK01 TW 009648]."

We note that you have provided additional information within the Acknowledgements Section that is not currently declared in your Funding Statement. Please note that funding information should not appear in the Acknowledgments section or other areas of your manuscript. We will only publish funding information present in the Funding Statement section of the online submission form.

Please remove any funding-related text from the manuscript and let us know how you would like to update your Funding Statement. Currently, your Funding Statement reads as follows:

"No"

Additionally, because some of your funding information pertains to [commercial funding//patents], we ask you to provide an updated Competing Interests statement, declaring all sources of commercial funding.

In your Competing Interests statement, please confirm that your commercial funding does not alter your adherence to PLOS ONE Editorial policies and criteria by including the following statement: "This does not alter our adherence to PLOS ONE policies on sharing data and materials.” as detailed online in our guide for authors <http://journals.plos.org/plosone/s/competing-interests>. If this statement is not true and your adherence to PLOS policies on sharing data and materials is altered, please explain how.

**Response: The authors declare this statement is true**

Please include the updated Competing Interests Statement and Funding Statement in your cover letter. We will change the online submission form on your behalf.

**Response: This has been completed.**

4. Please include your full ethics statement in the ‘Methods’ section of your manuscript file. In your statement, please include the full name of the IRB or ethics committee who approved or waived your study, as well as whether or not you obtained informed written or verbal consent. If consent was waived for your study, please include this information in your statement as well.

**Response: We have added the IRB number to the manuscript (bottom of page 9). We provide a discussion on our ethical considerations on page 10 to 11.**

Reviewers' comments:

Reviewer's Responses to Questions

**Comments to the Author**

Reviewer #1: Child mental Health Predictors among the Camp Refugees


My general comment: The manuscript has great potential to generate good scientific results in the area of traumatic stress and refugee mental health studies. However, the study’s objectives are not clear, a little confusing and hard to follow. Can the authors come up with hypotheses too.

**Response: All changes to the manuscript are highlighted in yellow. We have added a “Present Study” section in the manuscript to better illustrate the goals and objectives of the study. We have added three central research questions to this section and have chosen not to include a hypothesis. We hope these modifications are sufficient.**

Please see my brief specific comments below.

Abstract:
Objectives
I fail to understand the distinction between the following objectives
1.Describe the best predictors of Children’s depressive symptoms
2. Determine the variable importance of predictors of depressive ……
3. To provide “initial” what do the authors mean by “Initial”
-Please provide the statistical software used in the analysis

**Response: This has been addressed. Note that the statistical software was already mentioned in the manuscript.**

Generally, the abstract is confusing: The authors should bear in mind the structure of the abstract as follows.
a) Background/ introduction
b) Objective
c) Methods
d) Results
e) Conclusions and Recommendations

**Response: Thank you for the feedback. We have revised our abstract for clarity, however, we have chosen to remain with an unstructured/narrative abstract. We note that The journal allows for both abstract formats.**

The Background to the study is well written but brief. The authors could benefit from the following previous studies 1: <https://pubmed.ncbi.nlm.nih.gov/24478246/Journal> of traumatic stress, 27(1), 35–41. <https://doi.org/10.1002/jts.2189> (2) <https://doi.org/10.1080/20008198.2017.1283086https://www.ncbi.nlm.nih.gov/pmc/articles/PMC5328389/>

**Response: We have significantly expanded on our background and have provided much needed contextual information for the study. We hope these changes will be welcomed by the reviewers.**

It is not clear whether the authors are assessing the children on their personal experiences or the parent’s potentially traumatic experiences.

**Response: Thank you for this feedback! It is difficult to completely separate these two factors. In general, both of these factors play an important role in a child's mental health and we consider both. We have added on to the background section to better illustrate our central rationale undergirding the study, including the addition of a Present Study section. We hope these additions make our objectives more clear.**

Methods are described and written well.
Results: Have significant potential. However, since the objectives weren’t clear from the inception, the flow of the results poses difficulty to readability.

**Response: We have rewritten the objectives/hypotheses.**

Reviewer #2: The manuscript was technically good and sound. But there were some issues to be addressed and amendments. Please see the details at the PDF. Issues were given as comments on the PDF.

**Response: We have modified as the reviewer asked with a few exceptions. The style of abstract is different from the format given by the format suggested by the style template so no change was made. This is also the case with the limitations and recommendations. The comment in the introduction asking about the importance of the study is clearly stated by the lines highlighted in this section. Another comment asked about OfERR which the reviewer perhaps missed, as it was already included in the manuscript. The question at the beginning of the Data Collection section asked whether the seven year old data is represented for today, which we feel is not an appropriate question since this manuscript is focused on the analysis of the collected data, and not on the predictive validity of the data. There is another comment in this section that asks about the fraction which gave oral or written consent; the authors feel that this is irrelevant when it comes to the analysis, as both forms of consent are valid. There is a question on the reasons for only taking 4 male participants, but there is no reason for this aside from convenience sampling. This includes all the data collected.**

Why only 120 samples were measured? Is there any limitation? Does it exceeds minimal sample size? What is the number of adolescents of that camp? (See all comments at the manuscript PDF file)

**Response: Thank you for the feedback. We have included a power analysis (page 16) that indicates the sample size is acceptable. We hope this addition addresses questions related to sample size.**

Sincerely,

The authors
