# Supplementary material for: Child mental health predictors among camp Tamil refugees: Utilizing linear and XGBOOST models
Source: PLoS One. 2024 Sep 16;19(9):e0303632. doi: 10.1371/journal.pone.0303632 (PMC11404815; doi:10.1371/journal.pone.0303632)
Supplement: Attachment — Submitted filename: PONE-D-23-22224 with reviewer comments.pdf [file pone.0303632.s002.pdf]

PLOS ONE

Child Mental Health Predictors Among Camp Refugees: Utilizing Linear and XGBOOST Models

--Manuscript Draft--

**Manuscript Number:**	PONE-D-23-22224
**Article Type:**	Research Article
**Full Title:**	Child Mental Health Predictors Among Camp Refugees: Utilizing Linear and XGBOOST Models
**Short Title:**	Child Mental Health Predictors Among Camp Refugees
**Corresponding Author:**	Miriam Kuttikat<br>Virginia Commonwealth University School of Social Work<br>Richmond, Virginia UNITED STATES
**Keywords:**	Transmigration stressors, refugee mental health, refugee family functioning, machine learning
**Abstract:**	Research on migration has linked transmigration stress to deteriorated refugee mental health outcomes. However, much of this research focuses heavily on adult populations. Consequently, very little is known regarding the effects of the transmigration process on child mental health. This study examines the relationship between parental mental health and child mental health among refugees experiencing transmigration. We conducted in-person structured survey interviews with 120 parent-adolescent dyads living in the Trichy refugee camp in Tamil Nadu, India. Descriptive, multivariate analysis (hierarchical regression), and Machine Learning Algorithm (XGBOOST) were conducted to (1) describe the best predictors of child depressive symptoms as well as the levels of adjustment and family functioning, (2) provide initial tests of association between parental health and child well-being, and (3) determine the variable importance for predictors of child depressive symptoms. Results confirm parental mental health and child behavioral and emotional factors are significant predictors of child depressive symptoms. Although our linear model did not reveal a significant association between child mental health and family functioning, results from XGBOOST suggest high variable importance of family functioning in contributing to child depressive symptoms. Results amplify the urgency of mental health resources for both parents and children as well as parenting interventions inside refugee camps.
**Order of Authors:**	Muna Saleh
	Elizabeth Amona
	Miriam Kuttikat
	Indranil Sahoo
	David Chan
	Kyeongmo Kim
	Hannah George
**Additional Information:**	
**Question**	**Response**
**Financial Disclosure**<br><br>Enter a financial disclosure statement that describes the sources of funding for the work included in this submission. Review the submission guidelines for detailed requirements. View published research articles from *PLOS ONE* for specific	No

Powered by Editorial Manager® and ProduXion Manager® from Aries Systems Corporation

examples.<br><br>This statement is required for submission and **will appear in the published article** if the submission is accepted. Please make sure it is accurate.<br><br>**Unfunded studies**<br>Enter: *The author(s) received no specific funding for this work.*<br><br>**Funded studies**<br>Enter a statement with the following details:<br>• Initials of the authors who received each award<br>• Grant numbers awarded to each author<br>• The full name of each funder<br>• URL of each funder website<br>• Did the sponsors or funders play any role in the study design, data collection and analysis, decision to publish, or preparation of the manuscript?<br>• **NO** - Include this sentence at the end of your statement: *The funders had no role in study design, data collection and analysis, decision to publish, or preparation of the manuscript.*<br>• **YES** - Specify the role(s) played.<br><br>* typeset	
**Competing Interests**<br><br>Use the instructions below to enter a competing interest statement for this submission. On behalf of all authors, disclose any competing interests that could be perceived to bias this work—acknowledging all financial support and any other relevant financial or non-financial competing interests.<br><br>This statement is required for submission and **will appear in the published article** if the submission is accepted. Please make sure it is accurate and that any funding sources listed in your Funding Information later in the submission form are also declared in your Financial Disclosure statement.	No authors have competing interests

Powered by Editorial Manager® and ProduXion Manager® from Aries Systems Corporation

View published research articles from *PLOS ONE* for specific examples.<br><br>**NO authors have competing interests**<br><br>Enter: *The authors have declared that no competing interests exist.*<br><br>**Authors with competing interests**<br><br>Enter competing interest details beginning with this statement:<br><br>*I have read the journal's policy and the authors of this manuscript have the following competing interests: [insert competing interests here]*<br><br>* typeset	
**Ethics Statement**<br><br>Enter an ethics statement for this submission. This statement is required if the study involved:<br><br>• Human participants<br>• Human specimens or tissue<br>• Vertebrate animals or cephalopods<br>• Vertebrate embryos or tissues<br>• Field research<br><br>Write "N/A" if the submission does not require an ethics statement.<br><br>General guidance is provided below. Consult the submission guidelines for detailed instructions. **Make sure that all information entered here is included in the Methods section of the manuscript.**	IRB received for this study from Virginia Commonwealth University, Richmond, U.S.A. The approval I.D. number is HM20000475. Written consent form received from the participants of this study; All the demographic inofrmation were encrypted.

**Format for specific study types**<br><br>**Human Subject Research (involving human participants and/or tissue)**<br>• Give the name of the institutional review board or ethics committee that approved the study<br>• Include the approval number and/or a statement indicating approval of this research<br>• Indicate the form of consent obtained (written/oral) or the reason that consent was not obtained (e.g. the data were analyzed anonymously)<br><br>**Animal Research (involving vertebrate animals, embryos or tissues)**<br>• Provide the name of the Institutional Animal Care and Use Committee (IACUC) or other relevant ethics board that reviewed the study protocol, and indicate whether they approved this research or granted a formal waiver of ethical approval<br>• Include an approval number if one was obtained<br>• If the study involved *non-human primates*, add *additional details* about animal welfare and steps taken to ameliorate suffering<br>• If anesthesia, euthanasia, or any kind of animal sacrifice is part of the study, include briefly which substances and/or methods were applied<br><br>**Field Research**<br><br>Include the following details if this study involves the collection of plant, animal, or other materials from a natural setting:<br>• Field permit number<br>• Name of the institution or relevant body that granted permission	
**Data Availability**<br><br>Authors are required to make all data underlying the findings described fully available, without restriction, and from the time of publication. PLOS allows rare exceptions to address legal and ethical concerns. See the PLOS Data Policy and FAQ for detailed information.	Yes - all data are fully available without restriction

Powered by Editorial Manager® and ProduXion Manager® from Aries Systems Corporation

A Data Availability Statement describing where the data can be found is required at submission. Your answers to this question constitute the Data Availability Statement and **will be published in the article**, if accepted.<br><br>**Important:** Stating 'data available on request from the author' is not sufficient. If your data are only available upon request, select 'No' for the first question and explain your exceptional situation in the text box.<br><br>Do the authors confirm that all data underlying the findings described in their manuscript are fully available without restriction?	
**Describe where the data may be found in full sentences. If you are copying our sample text, replace any instances of XXX with the appropriate details.**<br><br>• If the data are **held or will be held in a public repository**, include URLs, accession numbers or DOIs. If this information will only be available after acceptance, indicate this by ticking the box below. For example: *All XXX files are available from the XXX database (accession number(s) XXX, XXX.).*<br>• If the data are all contained **within the manuscript and/or Supporting Information files**, enter the following: *All relevant data are within the manuscript and its Supporting Information files.*<br>• If neither of these applies but you are able to provide **details of access elsewhere**, with or without limitations, please do so. For example:<br><br>*Data cannot be shared publicly because of [XXX]. Data are available from the XXX Institutional Data Access / Ethics Committee (contact via XXX) for researchers who meet the criteria for access to confidential data.*<br><br>*The data underlying the results presented in the study are available from (include the name of the third party*	The data underlying th results presented in the study are available from Dr. Miriam Kuttikat, kuttikatm@vcu.edu

and contact information or URL).<br>• This text is appropriate if the data are owned by a third party and authors do not have permission to share the data.<br><br>* typeset	
Additional data availability information:	

Manuscript Click here to access/download;Manuscript;Manuscript.doc

Child Mental Health Predictors Among Camp Refugees: Utilizing Linear and XGBOOST Models

Muna Saleh[1]¶ , Elizabeth Amona[2]¶, Miriam Kuttikat[1]¶, Indranil Sahoo[2]¶, David Chan[3]¶, Kyeongmo Kim[1]¶, Hannah George[4]¶

School of Social Work, Virginia Commonwealth University, Richmond, Virginia, U.S.A.

Department of Statistical Sciences and Operations Research, Virginia Commonwealth University, Richmond, Virginia, U.S.A.

Department of Applied Mathematics, Virginia Commonwealth University, Richmond, Virginia, U.S.A.

Department of Anthropology,George Washington University, Washington D.C., U.S.A.

* Corresponding author
E-mail:Kuttikatm@vcu.edu

¶ These authors contributed equally to this work

ABSTRACT

Research on migration has linked transmigration stress to deteriorated refugee mental health outcomes. However, much of this research focuses heavily on adult populations. Consequently, very little is known regarding the effects of the transmigration process on child mental health. This study examines the relationship between parental mental health and child mental health among refugees experiencing transmigration. We conducted in-person structured survey interviews with 120 parent-adolescent dyads living in the Trichy refugee camp in Tamil Nadu, India. Descriptive, multivariate analysis (hierarchical regression), and Machine Learning Algorithm (XGBOOST) were conducted to (1) describe the best predictors of child depressive symptoms as well as the levels of adjustment and family functioning, (2) provide initial tests of association between parental health and child well-being, and (3) determine the variable importance for predictors of child depressive symptoms. Results confirm parental mental health and child behavioral and emotional factors are significant predictors of child depressive symptoms. Although our linear model did not reveal a significant association between child mental health and family functioning, results from XGBOOST suggest high variable importance of family functioning in contributing to child depressive symptoms. Results amplify the urgency of mental health resources for both parents and children as well as parenting interventions inside refugee camps.

INTRODUCTION

The United Nations High Commissioner for Refugees (UNHCR) reports that there were an estimated 103 million forcibly displaced persons worldwide in mid-2022, with 74% of refugees hosted in low-and-middle-income countries (LMIC) (UNHCR, 2022). Additionally, the United Nations International Children's Emergency Fund (UNICEF) estimates that 50% of the world's

refugees are children (2022). UNHCR reports that most refugees in need of resettlement–such as those living in refugee camps–are survivors of torture and violence.

Exposure to traumatic stressors has been previously linked to the development of psychopathology in youth, with earlier experiences most detrimental to a child's well-being (Fernando et. al., 2010). Those children undergoing migration firsthand become exposed to the dangers inherent in making the journey from a country experiencing a humanitarian crisis to the refugee camps, often in neighboring countries (Refugees International, 2022; Meda, 2017), and may experience isolation, dangerous living conditions, poverty, reduced family resources, and poor access to quality education and healthcare upon arrival. Given their vulnerability during displacement, youth are at exceptional risk for mental health problems caused by traumatic stressors (George, 2013; Driscoll et al., 2008; Fazel & Stein, 2002) encountered by both themselves and their caregivers.

Parents under duress and living in protracted statelessness may be particularly at risk for deterioration in mental health which may threaten parental coping and functioning which contribute to the intergenerational transmission of trauma (Gorge, 2010; Dalgaard & Montgomery, 2017; Timshel et al., 2017; Montgomery, 2008; Faizel et al., 2005). Taken together, these risks put displaced parents at elevated risk for impeded parenting resiliency and for transferring maladaptive coping models to their children (Akesson & Sousa, 2020). Similarly, young children may be deprived of the early development crucial to healthy brain development dependent on stable, psychosocial circumstances (Strouffe, 1997; Rogosch et al., 1995). Due to the complexity of conducting health research in refugee camps, very little research is devoted to studying the physical and mental health effects experienced in the transmigration process, and even less so on children.

Drawing on this research, we investigate how parental transmigration stress affects child depressive symptoms and how displaced families cope in these circumstances. The present study is guided by the following three objectives: (1) what are the best predictors of child depressive symptoms as well as the current levels of adjustment and family functioning; (2) provide tests of association between parental health and child well-being; and (3) determine the variable importance for predictors of child depressive symptoms among camp refugee youth. In meeting these objectives, we describe the relationships between parental mental health, child well-being, daily stress, and family functioning in the context of forced displacement.

METHODOLOGY

Setting

Over 125,000 Sri Lankan refugees currently live in India, after a civil war in their native country that resulted in a migration of refugees to different areas of the globe. Across three separate migration waves in 1984, 1999, and 2006, approximately 20,000 Sri Lankan refugees settled in Southern India. Currently, 90,000 of these migrants to India live in refugee camps. This research was done in partnership with the Organization for Eelam Refugee Rehabilitation, or OfERR, a non-profit group founded in 1984. The organization's mission is to help and provide resources to Sri Lankan refugees in India, which they do by working to ensure sustainable development for those in refugee camps. One such camp is in Trichy, a city in India's southern state of Tamil Nadu; the camp houses 1500 families. Recruitment and data collection at the Trichy camp lasted approximately one year, between June of 2014 to August of 2015. The institutional review board at the authors' university approved all practiced study procedures.

Sampling

The first and seventh authors, along with a team of 12 trained researchers, all female health workers and natives to Trichy, conducted interviews among 120 parent-adolescent dyads (120 parents and 120 adolescents) from the Trichy refugee camp. The research data collection team presented information about the study, such as inclusion criteria and notes about voluntary participation, at community events hosted by OfERR for the Sri Lankan refugee community. Stratified purposive sampling techniques were used to select participants in the study. Inclusion criteria for the study were 1.) Sri Lankan refugee status; 2.) Tamil ethnicity; 3.) participation of one parent-adolescent dyad in a family; and 4.) adolescents must have been between the ages of 13 and 18 at the time of data collection. A specific process was used to ensure a representative sample of 40 families from each subdivision of the refugee camp. First, a number between 1 and 1500 was randomly generated to establish a starting point. Next, every 8 th household was selected for the study. The research team visited 180 homes, after which 120 families, with children and parents living together, contacted them to inform the team of their interest in participating in the study.

Data Collection

The data collection started on July 12, 2016 and completed on August 14, 2017. Based on the individual preference of the potential participant, the research team described the study and performed the screening process using phone calls in Tamil or English. Selected participants were then notified to arrange a time and date to sign the Tamil-translated consent form and partake in a face-to-face interview. All participating parents signed a written informed consent for themselves and all children signed a written assent form for themselves to participate in the study and voluntary adolescent participation was verified during screening by confirming with

both the parent and adolescent of the dyads. To deal with potential ethical issues concerning informed consent from adolescents, the research team thoroughly explained the study in simple language so the data collection methods would be understood and asked the adolescent participants what they felt comfortable sharing; adolescents were not required to answer questions they did not want to respond to. In addition to ensuring informed consent, the research team conducted MINI exams to assess participants' mental and cognitive status. When more than one adolescent in the same age group from one family volunteered to participate, the research team assessed the child's MINI exam and coordinated with parents and health workers to select one child based on need.

Refugees who have experienced negative interactions with authorities and/or foreign cultural traditions may be suspicious of written consent forms. The option of oral consent was provided in these cases, and clear procedures on how to obtain and record verbal consent were given to the research team. Verbal consent was always gained from adolescents. In situations in which gatekeepers had been involved in participant recruitment, it was necessary that research respondents understood their right to refuse participation at any stage in the research process. This refusal would not affect available services or level of care provided. The anonymity of the participants was treated with extreme consideration, given that an agency/service had referred them. The only job of the research team was to collect data, which was then organized, with each parent and adolescent participant receiving assigned codes. Demographic information from the participants was stored at the referral agency and was accessible only to the primary researcher. The research team was instructed on the importance of building relationships with camp residents, building rapport and establishing trust. The team made sure that the relationships were equally important to adolescents. The relationships were aided by previous

collaboration between researchers and camp residents on a few pilot studies in the Trichy camp.

These prior research experiences helped the refugees verify the research team's true intentions and trust the absence of coercion and power imbalance. In the current study, rapport was maintained through research and regular attendance at community events sponsored by OfERR; these visits also acted as data collection. Researchers were also able to spend time with adolescents and parents outside of official data collection procedures, getting to know them on a deeper level and engaging with their interests and experiences. This approach is demonstrably useful, as evidenced by the excitement adolescents almost always showed when they saw the first author. The research team was also able to obtain social, linguistic, and facial interaction knowledge with refugee families at the Trichy camp, which helped unwanted interactions remain avoided. Regardless of these precautions and attempts, it is acknowledged that this process may not always reflect autonomous agreement to participate in facets of the study.

At the convenience of the research participants, all interviews were conducted in the OfERR office. Parents and adolescents were interviewed separately in different rooms. When it came to interviews between researchers and adolescents, the research team was eager to ensure that the adolescents were not only comfortable speaking to them, but also that the adolescents were able to see them as neither a 'teacher' nor a 'parent', to guarantee the adolescents would feel safe sharing their experiences. This detail was particularly important given that young children typically provide socially desirable and/or confirmatory responses to please adults. If a male was being interviewed, an elder woman stood a few feet away from the door to ensure the researchers' safety, as is custom in the participants' tradition. After the conclusion of an

interview, participants engaged in a debriefing session with the researcher, in which they could discuss questions or concerns. After the interview, every family was compensated 1000 Indian Rupees for their participation, given to the parents as per custom. Each interview lasted approximately two hours with three five-minute breaks.

Measurement

Child Depressive Symptoms: The child mental health variable consisted of one variable: child depressive symptoms. Child depressive symptoms were measured using the 27-item Child Depression Inventory (CDI) (Kovacs, 1992). As one of the most widely used and best studied scales in measuring child depressive symptoms (Kazdi[illegible] 1989; Slodnjak et al., 2002), the CDI captures affective, cognitive and behavioral symptomatology consistent with depression. Participants were asked to indicate items that describe them best in the past two weeks. Scores were summed for a total score of depression. Scores range from 0-54 with higher scores indicating higher depression. Although cutoff scores for both clinical and nonclinical settings have been suggested, they are culture-bound, and thus not used in this study. This decision was made due in part to the cultural norms with this study population and for the purpose of this research, which is to identify relationships among the key study variables and not focus on identifying depression. Cronbach's alpha for this sample was .772, indicating good reliability.

Parental Mental Health: Parental mental health was measured using the Brief Symptoms Inventory Scale (BSI) (Derogatis, 1983). The BSI consists of a total of nine dimensions, however, this study utilized four domains including depressive symptoms (six items), hostility (five items), anxiety symptoms (six items), and somatization (seven items). Using a 5-point Likert Scale ranging from 1 (not at all) to 5 (extremely), participants were asked how often they

experienced an item in the last seven days. Responses were then summed to arrive at a total score, with higher scores indicating higher levels of mental distress. BSI scores ranged from 0-79, with an average score of 27.3. Reliability was assessed for each subscale. Cronbach's alpha was calculated at .700 for the somatization subscale, .818 for the depressive symptoms, .776 for anxiety symptoms, and .664 for hostility subscales.

Child Daily Stressors: Child daily stressors were assessed using the 29 item Children's Daily Stressors Scale (CDSS) (Miller et al., 2009). This scale was specifically developed for Sri Lankan youth who have been exposed to war and trauma. The purpose of the scale is to examine the contributions of war, disaster exposure and daily stressors on the mental health of children (Miller et al., 2009). It includes three subscales: depression (ten items), inter-parental conflict (4 items), and abuse (6 items). On a 3-point Likert Scale ranging from 1-3, participants were asked how stressful they currently found each item (such as inadequate water/housing, physical abuse by authority figures, and parental substance use) to be. Responses included "very stressful", "somewhat stressful", and "not stressful". Higher scores indicated higher levels of daily stress. Cronbach's alpha was calculated to be .848, indicating high reliability.

Family Functioning: Two variables comprise the family functioning variable: Quality of Parenting and Family Cohesion. Quality of parenting was measured using a shortened version of the Child Report of Parent Behavior Inventory (CRPBI). Specifically, the 20-item Acceptance Subscale (Raskin et al., 1971; Schaefer, 1965) was used to assess children's perceptions of parental acceptance and quality of caregiving. Adolescent participants were asked a series of parenting-specific behaviors (e.g., "understands your problems and your worries", "enjoys doing things with you") to rate as either "A lot Like", "Somewhat like", or "Not like" their parents. Higher total scores of parental qualities signify lower levels of parental quality.

Reliability for this scale was calculated at .843. Family Cohesion was assessed using a shortened, 18-item version of the Child Self-Report of Family Inventory Scale (SFI; Beavers et al., 1985). The full, 36-item SFI assesses family competency and comprises six domains, including Health/Competence, Conflict, Cohesion, Leadership, Communication and Emotional Expressiveness (Goodrich et al., 2012). The Communication and Cohesion domains were used in this study. Cronbach alpha for this sample was .864, indicating high reliability.

Child Sleep Quality: To assess the presence of sleep problems among the adolescent sample, researchers administered the Pittsburgh Sleep Quality Index. The scale includes a total of 15 items pertaining to respondent's sleeping patterns in the past two weeks. Cronbach's alpha for this sample was calculated at .811, indicating high reliability. Higher total scores indicate poor sleep quality.

Child Total Strengths and Difficulties: To assess adolescent behavioral and emotional problems, the Child Report of Total Strengths and Difficulties Questionnaire (SDQ) (Goodman, 1997) was used. The SDQ is widely used across the globe for children between the ages of 11 and 17 (Goodman et al., 2010). The scale comprises a total of 25 items and five subscales including emotional, conduct problems, hyperactivity/inattention, peer relationship problems and prosocial behaviors. Cronbach's alpha was .746 for this sample, indicating good reliability.

DATA ANALYSIS

To test the associations and predict the variables contributing to child depressive symptoms, we use both linear and nonlinear methods, namely, hierarchical regression and XGBOOST. Utilizing these methods in conjunction allows us to test the variable importance and help overcome limitations presented by the data. While linear models allow us to test model

significance, Mean Square Errors, adjusted R^2, and change in R^2 ($R^2\Delta$), they are limited in model flexibility and explaining variable importance when predicting the outcome variable. Thus, we adopted a supplemental method with the ability to explain variable importance of the predictors and perform concurrent cross validation. Using XGBoost, we illustrate which variables are important in predicting child depressive symptoms, and also examine the dependency of each predictor.

Linear Model

SPSS version 28 was used to conduct bivariate and multivariate (specifically hierarchical regression) analyses. We conducted a hierarchical regression analysis to test predictors for child depressive symptoms. In step one, child specific variables were entered into the model (child sleep quality, child strengths and difficulties and child daily stressors). In step two, parent-specific mental health variables were entered into the model (parent mental health). In the final step, family functioning variables (parenting quality and family cohesion) were added to the model.

Extreme Gradient Boosting (XBOOST)

In addition to the linear model, we implemented a nonlinear machine learning algorithm, namely Extreme Gradient Boosting (XGBoost). XGBOOST provides a flexible framework for modeling child depressive symptoms using the six predictor variables. The XGBoost algorithm is a regularized tree boosting method originally proposed by Chen & Guestrin (2016). This method has achieved excellent predictive performance in many fields, and is considered extremely suitable for the statistical analysis of big data (Wang et al., 2022). The predictive model for child depressive symptoms was fitted using the XGboost method and the variable importance of the six predictors were calculated concurrently. Lastly, SHapley Additive

exPlanations (SHAP) summary plots, proposed by Lundberg et al., 2020, were used to visually interpret the impact of these predictors on the response. The analysis was done in R software using "SHAPforxgboost" package (Yang & Allan, 2020).

XGBoost Model Setup

The data analysis was performed on open-source RStudio version 4.2.0 using packages "xgboost," "caret," "mlr," "ROSE," "DMwR," and "ggplot2". We split the dataset into 80% training data, and 20% testing data. The model was fitted on the training dataset, and then fine-tuned through the boosting procedure using a k-fold cross-validation technique until convergence. For details on the algorithm, see Chen and Guestrin, 2016.

SHapley Additive exPlanations (SHAP) Method

The Shapley value, coined by Shapley (Roth, 1988; Shapley, 1953), is a game theoretic approach of assigning credit to 'players' in a fair manner depending on their contribution to the total output of a 'game'. Quantifying Shapley values for features in regression or machine learning models is a recent development, first implemented by Lipovetsky and Conklin (2001), Cohen et al. (2005), and later developed by Štrumbelj and Kononenko (2014). This work led to the development of the SHAP package for Python (Lundberg and Lee, 2017). Nowadays, Shapley values are widely used in applied machine learning areas such as: medicine (Ibrahim et al., 2020; Smith & Alvarez, 2021), chemistry and pharmaceutical research (Rodríguez-Pérez and Bajorath, 2019, 2020), economics (Antipov and Pokryshevskaya, 2020), and environmental research (Wikle et al., 2022).

Shapley values quantify the contribution of each predictor variable to the prediction, as compared to a baseline average prediction. For linear models, these values are just the values of each feature multiplied by their corresponding weights. However, the quantifications are

complex for non-linear models. To connect the game-theoretic context to model based predictions, consider the 'game' as a prediction task. The corresponding 'gain' is the actual prediction after including a particular predictor minus the model's average prediction, and the 'players' are the predictors that collaborate to receive the gain (i.e., predict a certain value). For technical details on Shapley values, including mathematical formulation, see Lundbeg et al., 2018.

RESULTS

Sample Characteristics and Descriptive Statistics

Descriptive statistics for all key variables are presented in Table 1. Parents were overwhelmingly female (96%) and married (87%). Average age for parents was 38 years old. Average number of children per parent was about three (2.86), and average time spent in the camp was 22.67 years. Average age of child participants was 14.23 years, with average score for child depressive symptoms at 11.61 (*SD* = 5.54). Mean scores for total parent BSI scores was 27.28 (SD=6.4) and ranged from 0 to 79.; average scores for child daily stressors were 18.42 (*SD* = 16.34); child depressive symptoms mean score was 11.6 (SD=5.5); average child sleep quality was 5.81 (SD=5.21). Mean levels of parenting quality was 48.41 (SD=13.10) and family cohesion at 55.98 (*SD* = 13.21).

Table 1: Descriptive statistics: Demographic & Key Variables

	Frequency (%)	M	SD	Mode	Range	Median	Missing
Demographics							
**Parent**							
Male	4 (3.4)						
Female	115 (95.8)						
Age	--	38.00	6.62	35	36	37	
Leng.in camp	--	22.67	4.98	24.00	31.00	24.00	
Married	100 (87)						

Widowed	10 (8.3)						
Separated	5 (4.2)						
# Children	--	2.86	.963	3.00	5	3.00	
Employed	38 (31.7)						
Unemployed	79 (65.8)						
**Child**							
Age		14.23	1.64	13.00	6.00	14.00	
Sex							1
Male	43(37%)						
Female	73(63%)						
Mental Health							
Child Depress		11.6	15.5		26	11	2
Parent BSI		27.3	18.5		79 (0-79)	25	
Child Daily stress		18.4	16.3		55(0-55)	11.7	1
Total Difficulties		10.2	5.4		22 (2-24)	10	
Family Functioning							
Parenting Quality		48.1	7.6		33.7(26.3-60)	50	
Family cohesion		56.0	13.3		52.3(26.5-78.8)	57.3	
Child Sleep Quality		5.8	5.2		26(0-26)	5	2

n=117

Linear Analysis

The data met the assumptions for regression analysis. The residuals were independent, and there was no concern for multicollinearity, as supported by the correlation matrix table and the VIF scores for each variable (not shown here). Additionally, the variables were all linear with the outcome variable, and the assumption for homoscedasticity was met. Hierarchical regression analysis was conducted to examine the main effects of parental mental health, child-specific variables, and family functioning variables on child mental health. Results from the regression analysis, including standardized regression coefficients (β), R^2 and $R^2\Delta$ are summed in Table 2. Results from bivariate correlation test and previous literature on child mental health were used to determine the variables included in the model. In the first model, 29.8% of the variance in child

depressive symptoms was accounted for, $R = .546$, $F(3, 107) = 15.127$, $p < .001$. In the second model, 32.8% of the variance in the response variable was accounted for, $R = .573$, $F(4, 106) = 12.939$, $p < .001$. In the last model, 34.6% of the variance in child depressive symptoms was accounted for by the variables included in the model, $R = .588$, $F(6, 104) = 9.177$, $p < .001$.

Results (Table 2) indicated two statistically significant predictors of child depressive symptoms in the model while keeping other predictors fixed, we see that every unit of increase in parent mental health predicted a .171 increase in child depressive symptoms ($p = .037$). Additionally, every unit increase in child total difficulty and strengths predicted a .441 increase in child depressive symptoms ($p < .001$). Parenting quality was not significant (p=.097).

Table 2: Hierarchical Regression Results

Hierarchical Regression Results	B	95% CI	SE	β	R^2	ΔR^2
Constant	5.678***	[3.529, 7.83]	1.084		.298	
Child Sleep Qual	-.022	[-.202, .158]	.091	-.021		
Child daily stress	.009	[-.044, .061]	.026	.028		
Diff & Strength	.550***	[.376, .723]	.088	.553		
Constant	4.526***	[2.170, 6.883]	1.188		.328	.030
Child Sleep Qual	-.008	[-.186, .169]	.090	-.008		
Child daily stress	.009	[-.043, .060]	.026	.027		
Diff & Strength	.516***	[.343, .690]	.087	.519		
Parent BSI	.053**	[.005, .101]	.024	.177		
Constant	11.285*	[2.567, 20.004]	4.396		.346	.018
Child Sleep Qual	-.033	[-.212, .147]	.091	-.032		
Child daily stress	.005	[-.050, .059]	.028	.014		
Diff & Strength	.438***	[.240, .636]	.100	.441		
Parent BSI	.051**	[.003, .099]	.024	.171		
Parenting Quality	.000	[-.258, .022]	.035	.001		
Family Cohesion	-.117*	[-.069, .070]	.070	-.163		

* $p<.05$, ** $p<.01$, *** $p<.001$

XGBOOST Results

Only seven iterations were needed to obtain the final model (with the smallest mean square error), and this final model was used to understand the variable importance of the predictors. Below (Table 3) is the result detailing the variable importance of the model with 6 predictors. Note that these 6 predictors were also used for the linear model analysis.

Table 3: Variable Importance for the fitted XGBoost model

Feature	Gain
Child Strength & Difficulties	43%
Sleep Quality	24%
Child Daily Stressors	13%
Parenting Quality	8%
Family Cohesion	8%
Parent Mental Health	3%

Variable importance shows the relative contribution of each predictor to model accuracy, and is measured by ‘Gain’. In Table 3, each feature and their corresponding Gain scores are presented. Gain values are similar to the % of variance explained by each predictor in the model. In our analysis, child strength and difficulties were the most important variable in predicting child depressive symptoms with the highest variable importance of about 43%. Child sleep quality also contributed significantly to the child's depressive symptoms since the Gain value (about 24%) was second highest, followed by child daily stressors score with Gain value of about 13%. In addition, family cohesion and parenting quality each contributed about 8% to the prediction accuracy of child depressive symptoms.

Finally, the SHAP summary plot is used to explain the effect of each individual predictor on the response. Note that high Shapley values means that the effect was severe and low Shapley

means that the effect is not severe. Figure 1 shows the SHAP summary plot that explains the effect of the six predictors on the response (child depressive symptoms).

Figure 1: SHAP values (impact on model output)

Figure 1 shows that the child total strength and difficulties scores has a high positive effect on child depressive symptoms, therefore child depressive symptoms would increase as the child total strength and difficulties scores increased. Furthermore, parenting quality, sleep quality, child daily stressor and family cohesion have moderately high positive effects on child depressive symptoms. This means that depressive symptoms in children could worsen if parents have negative behavior towards them, if the family cohesion is poor or if the children do not have good sleep. Lastly, parent mental health had a moderate positive effect on the child's depressive symptoms, thus the child's depressive symptoms would moderately increase if parents had poor mental health. Also, note that the values in Figure 1 are not the variable importance percentages, but rather the means associated with each variable and hence are different from the values shown in Table 3.

DISCUSSION

The combined results from the linear and nonlinear analyses give us predictive models for child depressive symptoms and identify parental mental health, child total strength and difficulties and family functioning variables as the key predictors. These results are promising as they begin to explore potential factors associated with the mental health of refugee children. The nature of living in a refugee camp is extremely stressful, and is often accompanied by a lack of material resources, given that many refugees flee their home countries abruptly (Silove et al., 2017). In addition to forced displacement, Sri Lankan refugee families have witnessed traumatic and violent events and have undergone a great deal of loss (Silove, 1999). Refugees often come

to refugee camps with high levels of pre migration trauma (George, 2010) that devastates mental and physical wellbeing and adaptive coping.

Upon arrival to the camps, refugees must prepare to begin the process of living in displacement for an undetermined period of time and grapple with the traumatic and daily stressors associated with life in protracted displacement. Such challenges include disruptions in protective and adequate parenting styles and family structures that are often crucial in cultural child rearing practices, and the emergence of intergenerational conflicts in the context of liminality. Such experiences have great potential to disrupt the mental health of most people, let alone families with both extensive collective and individual trauma histories, and a battered resource reserve. It may be appealing to conclude that given these circumstances, we are limited in influencing the adverse exposure to distressing experiences for youth experiencing transmigration. Fortunately, results of this study begin to challenge this assumption. Although parents may experience impeded mental health in the context of transmigration (reference removed for blind review, 2022), in targeting child psychological distress, it may be worthwhile to focus interventions on improving family functioning as well as providing resources to address the social and emotional problems adolescents face.

Previous literature on parenting inside refugee camps have underscored the need for parenting resources inside refugee camps (El-Khani et al., 2016; reference removed for blind review). In addition to grappling with psychological adjustments, parents must also learn how to navigate complex relationships with their children in distressing environments. As parents learn to adapt to new environments, their ability to respond to their children may also shift (Williams, 1990). Prior to displacement, families may have had the financial, material, cultural and personal resources to carry out parenting roles effectively, which become depleted or deteriorated after

initial displacement occurs. Further, refugee camps are often overcrowded, under-resourced, and unprepared to respond to the psychosocial needs of the families contained in them. For families faced with daily stressors, they may not have the capacity or luxury to fully engage in reflective parenting practices (George & Jettner, 2016).

Our nonlinear model also revealed a significant association between sleep quality and child depressive symptoms. This makes intuitive sense, since when a child is depressed, sleep quality often becomes compromised. Even though this effect is moderate and complex to quantify, a possible intervention to help improve sleep in this population may be worthwhile.

There are a few notable limitations in this study. First, the measurements used may not be accurate in fully capturing the constructs under study in this paper. Specifically, only the Children's Daily Stressors scale was developed for use with Sri Lankan populations. Even then, the initial scale was developed with a sample of youth not experiencing transmigration, nor externally displaced (Miller, K., 2009). Next, there may be another construct that may better explain the mental health outcomes of youth experiencing transmigration, namely a transmigration stress construct. Identifying the specific stressors associated with displacement and contributing to poorer mental health for children is important for this particular population. Displaced refugees in camps may be more vulnerable to encounter traumatic stressors than perhaps other refugees who have either resettled, or have not left their home country (George, 2012). The acculturative stress, coupled with transmigration stress may contribute to mental health uniquely, and without proper measurement tools, it is unlikely that we may know the unique experiences of displaced refugee adolescents. This challenge of assessing specific issues with this population also leads to the difficulty of explaining in detail the variations seen in both models.

In summary, this study began to theorize the relationships among parental mental health, family functioning and child depressive symptoms in the context of forced displacement. Findings provide support that in the context of transmigration, family functioning may become significantly deteriorated, and may result in declines to parental mental health and child mental health outcomes. Findings underscore the need to develop and implement resources that build upon parenting capacities and that improve parental efficacy. Other key implications include the urgency to provide mental health interventions for parents in camp settings, to address their mental health needs.

ACKNOWLEDGEMENT

This work was supported by National institute of Health Fogarty International [grant numberK01 TW 009648].

REFERENCES

Akesson, B., Sousa, C. Parental Suffering and Resilience Among Recently Displaced Syrian Refugees in Lebanon. *Journal of Child and Family Studies.* 2020; 29:1264–1273. https://doi.org/10.1007/s10826-019-01664-6

Antipov, E. A., & Pokryshevskaya, E. B. Interpretable machine learning for demand modeling with high-dimensional data using gradient boosting machines and shapley values. *Journal of Revenue and Pricing Management.* 2020; 19(5): 355–364.

Armstrong, M., Birnie-Lefcovitc, S., and Ungar, M. (2005). Pathways Between Social Support, Family Well-Being, Quality of Parenting, and Child Resilience: What We Know. *Journal of Child and Family Studies*. 2005;14: 269–281. https://doi.org/10.1007/s10826-005-5054-4

Beavers, W. R., Hampson, R. B., & Hulgus, Y. F. (). Commentary: The Beavers systems approach to family assessment. *Family Process.* 1985; 24(3):398–405. https://doi.org/10.1111/j.1545-5300.1985.00398.x

Betancourt, T., Barnhart, D., Brennan, R., Murray, S., Yousafzai, A., Farrar, J., Godfroid, K., Bazubagira, S., Rawlings, L., Wilson, B., Sezibera, V., Kamurase, A. Promoting parent-child relationships and preventing violence via home-visiting: a pre-post cluster-randomized trial among Rwandan families linked to social protection programs. *BMC Public Health.* 2020; 20: 621. https://doi.org/10.1186/s12889-020-08693-7

Betancourt, T., Ng, L., Kirk, C., Brennan, R., Beardslee, W., Stula, S., Mushashi, C., Nduwimana, E., Mukunzi, S., Nyirandagijimana, B., Kalisa, G., Cyamatare, F., and Sezibera, V. Family-Based Promotion of Mental Health in Children Affected by HIV: A Pilot Randomized Controlled Trial. *Journal of Child Psychology& Psychiatry*. 2017; 58(8): 922–930. https://doi:10.1111/jcpp.12729

Betancourt, T., Abdi, S., Ito, B., Lilienthal, G., Agalab, N., & Ellis, H. We left one war and came to another: Resource loss, acculturative stress, and caregiver–child relationships in Somali refugee families. *Cultural Diversity and Ethnic Minority Psychology*.2015; 21(1): 114-125. https://www.ncbi.nlm.nih.gov/pubmed/25090142

Bradley M. *Refugee Repatriation: Justice, Responsibility and Redress*. Cambridge, UK: Cambridge University Press. 2013.

Buysse, D., Reynolds, C., Monk, T., Berman, S., & Kupfer,D. The Pittsburgh sleep quality index: A new instrument for psychiatric practice and research, Psychiatry Research. 1989; 28 (2):193-213. https://doi.org/10.1016/0165-1781(89)90047-4.

Cohen, S., Dror, G., & Ruppin, E. Playing the game of feature selection. *Proceedings of the 19th International Joint Conference on Artificial Intelligence.* 2005; August:1-8. https://www.researchgate.net/profile/Eytan-Ruppin/publication/228966610_Playing_the_game_of_feature_selection/links/0fcfd505c00fce8801000000/Playing-the-game-of-feature-selection.pdf.

Dalgaard, N. & Montgomery, E. "The transgenerational transmission of refugee trauma: family functioning and children's psychosocial adjustment". International Journal of Migration, *Health and Social Care.* 2017; *13*(3): 289-301. https://doi.org/10.1108/IJMHSC-06-2016-0024

Derogatis, L. R., & Melisaratos, N. The Brief Symptom Inventory: An introductory report. *Psychological Medicine.* 1983; *13*(3): 595–605. https://doi.org/10.1017/S0033291700048017

Driscoll, A. K., Russell, S. T., & Crickett, L. J. Parenting styles and youth well-being across immigrant generations. *Journal of Family Issues.* 2008; *29:*185-209. https://doi.org/10.1177/0192513X07307843

El-Khani, A., Ulph, F., Peters, S., & Calam, R. Syria: refugee parents' experiences and need for parenting support in camps and humanitarian settings. Vulnerable Children and Youth Studies. 2016; 13(1): 19-29. DOI: 10.1080/17450128.2017.1372651

Fazel, M., Wheeler, J., & Danesh, J. Prevalence of serious mental disorder in 7000 refugees resettled in western countries: a systematic review. The Lancet. 2005; 365 (9467): 1309-1314, https://doi.org/10.1016/S0140-6736(05)61027-6.

Fazel M & Stein A. The mental health of refugee children. Archives of Disease in Childhood. 2002; 87(5):366–370. doi: 10.1136/adc.87.5.366.

Fernando GA, Miller KE, Berger DE. Growing pains: the impact of disaster-related and daily stressors on the psychological and psychosocial functioning of youth in Sri Lanka. *Child Development.*2010*;* 81(4): 1192-210. doi: 10.1111/j.1467-8624.2010.01462.x.

George, M. & Jettner, J. Migration stressors, psychological distress and family - A Sri Lankan Tamil refugee analysis. *Journal of International Migration and Integration.* 2016;17 (2): 341-353. doi: org/10.1007/s12134-014-0404-y

George, M. Sri Lankan Tamil refugee migration experiences: A qualitative analysis. *International Journal of Culture and Mental Health*.2013; *6*(3): 170-182. doi: org/10.1080/17542863.2012.681669

George, M. Migration traumatic experiences and refugee distress: Implications for social work practice. *Clinical Social Work Journal.* 2012; *40*(4): 429-437. doi: org/10.1007/s10615-012-0397-y

George, M. The roles, strengths, and challenges of health educators in Toronto hospitals. *The International Journal of Interdisciplinary Social Sciences*, 2010; *5*(1): 445-456. doi: org/10.18848/1833-1882/CGP/v05i01/53083

Goodman, A. The Strengths and Difficulties Questionnaire: a research note. Journal of Child Psychology and Psychiatry.1997; 38: 581-586.DOI: 10.1111/j.1469-7610.1997.tb01545.x

Goodman, A., Lamping, D., Ploubidis, GB. When to Use Broader Internalising and Externalising Subscales Instead of the Hypothesised Five Subscales on the Strengths and Difficulties Questionnaire (SDQ): Data from British Parents, Teachers and Children. *J Abnormal Child Psychology*. 2010; *38* (8): 1179-91. DOI: 10.1007/s10802-010-9434-x

Goodrich, K. M., Selig, J. P., & Trahan, D. P. The Self-Report Family Inventory: An Exploratory Factor Analysis. *Measurement and Evaluation in Counseling and Development*. 2012; 45(4): 245–256. https://doi.org/10.1177/0748175612449173

Hutchings, J., & Lane, E. Parenting and the development and prevention of child mental health problems. *Current Opinion in Psychiatry.* 2005*;* 18(4): 386–391. https://doi.org/10.1097/01.yco.0000172056.63401.e0

Hynie, M. The Social Determinants of Refugee Mental Health in the Post-Migration Context: A Critical Review. *The Canadian Journal of Psychiatry*. 2017; *63*(5): 297–303. https://doi.org/10.1177/0706743717746666

Ibrahim, L., Mesinovic, M., Yang, K.-W., & Eid, M. A. Explainable prediction of acute myocardial infarction using machine learning and Shapley values. *IEEE Access*. 2020; 8: 210410–210417.

Kazdin, A.E. Childhood depression. *Journal of Child Psychology and Psychiatry.* 1990; 31:121–160. doi: 10.1111/j.1469-7610.1990.tb02276.x.

Kirmayer, L., Narasiah, L., Munoz, M., Rashid, M., Ryder, A., Guzder, J., Hassan, G.,Rousseau, C., Pottie, K. Common mental health problems in immigrants and refugees: general approach in primary care. *Canadian Medical Association Journal.*2011*;*183(12),959-67.

Kovacs M. *The Children's Depression Inventory (CDI) manual North Tanawanda.* New York, NY: Multi-Health Systems. 1992. https://doi.org/10.1002/9781118625392.wbecp419

Kuttikat, M., George, H.& George, D. Proverbs, and gender: The interface between proverbs and human relations in a refugee camp. *International Journal of Social Science Studies.*2022*;* 10 (6): November. DOI: https://doi.org/10.11114/ijsss.v10i6.5755

Lipovetsky, S., & Conklin,M. Analysis of regression in game theory approach. *Applied Stochastic Models in Business and Industry*. 2001;17(4): 319–330.

Lundberg, S. M., Erion, G., Chen, H., DeGrave, A., Prutkin, J., Nair, B., Katz, R., Himmelfarb, J., Bansal, N., & Lee, S. From local explanations to global understanding with explainable AI for trees" *Nature machine intelligence.* 2020;2(1): 56-67. https://doi.org/10.1038/s42256-019-0138-9

Lundberg, S.M., Nair, B., Vavilala, S., Horibe, M., Eisses, M., Adams, T., Liston, D., Low, D., Newman, S., Kim, J., & Lee, S. Explainable machine-learning predictions for the prevention of hypoxaemia during surgery. *Nature Biomedical Engineering*. 2018; 2: 749–760. https://doi.org/10.1038/s41551-018-0304-0

Lundberg, S. M. & Lee, S. A unified approach to interpreting model predictions. *Advances in neural information processing systems*. 2017;30.

Lundberg, S. M., Erion, G. & Lee, S. Consistent individualized feature attribution for tree ensembles. *arXiv preprint.* 2018*; arXiv:1802.03888.* https://doi.org/10.48550/arXiv.1802.03888

Meda, L. A Journey Without Planned Destination: Traumatic Transmigration Experiences of Refugee Children. *Int. Migration & Integration*. 2017;18: 131–142. https://doi.org/10.1007/s12134-016-0477-x

Molnar, Christoph. Intrpretable machine learning: A guide for making black box models explainable. Lean Publications; 2018. *Available from: https://christophm. github. io/interpretable-ml-book.*

Montgomery,E. Long-term effects of organized violence on young Middle Eastern refugees' mental health. *Social Science & Medicine*. 2008; 67(10), 1596-1603. https://doi.org/10.1111/j.1365-2214.2008.00935_1.x

Miller K, & Rasco L. *The Mental Health of Refugees: Ecological Approaches To Healing and Adaptation*. Taylor and Francis. 2004.

Miller KE. Refugee children. In: R. Schweder (Ed.), *The University of Chicago Companion to the Child*. Hyde Park, IL: University of Chicago Press; 2009: 812-813.

OfERR. Organization for Eelam Refugee Rehabilitation. 2022; Available from: https://oferrindia.org/

Ponnet, K. Financial stress, parent functioning and adolescent problem behavior: An actor–partner interdependence approach to family stress processes in low-, middle-, and high-income families. *Journal of Youth and Adolescence.* 2014;43 (10): 1752–1769. https://doi.org/10.1007/s10964-014-0159-y

Refugees International. Nowhere to Run: Eritrean Refugees in Tigray. 2022; Available from: https://www.refugeesinternational.org/reports/2022/3/1/nowhere-to-run-eritrean-refugees-in-tigray

Rodríguez-Pérez, R., & Bajorath, J. Interpretation of compound activity predictions from complex machine learning models using local approximations and Shapley values. *Journal of Medicinal Chemistry*. 2019; 63(16): 8761–8777.

Rodríguez-Pérez, R., & Bajorath, J. Interpretation of machine learning models using Shapley values: Application to compound potency and multi-target activity predictions. *Journal of Computer-Aided Molecular Design*. 2020; 34(10): 1013–1026.

Rogosch, F., Cicchetti, D., & Aber, L. The role child maltreatment in early deviations in cognitive affective processing abilities and later peer relationships. *Development and Psychopathology*. 1995; 7:591- 609. https://doi.org/10.1017/S0954579400006738

Roth, A. E. The Shapley value: Essays in Honor of Lloyd S. Shapley. Cambridge University Press.1988.

Schaefer, E.S. Children's Report of Parental Behavior: An Inventory. *Child Deviance.* 1965;36: 413-424. https://doi.org/10.2307/1126465

Schweitzer, R., Melville,F., Steel, Z., & Lacherez. P. Trauma, post-migration living difficulties, and social support as predictors of psychological adjustment in resettled Sudanese refugees. *Australian New Zealand Journal of Psychiatry*. 2006; 40(2):179-87. doi: 10.1080/j.1440-1614.2006.01766.x.

Shapley, L. S. A value for n-person games. In H. Kuhn & A. Tucker (Eds.), *Contributions to the theory of games. The Annals of Mathematics.* 1953;28: 307–317. Princeton University Press.

Silove, D. The Psychosocial Effects of Torture, Mass Human Rights Violations, and Refugee Trauma. *The Journal of Nervous and Mental Disease*. 1999; 187(4): 200-207. doi: 10.1097/00005053-199904000-00002.

Silove, D, Ventevogel, P, Rees, S. The contemporary refugee crisis: an overview of mental health challenges. *World Psychiatry*. 2017;16:130–139. doi: 10.1002/wps.20438.

Slodnjak, V., Kos, A., & Yule, W. Depression and Parasuicide in Refugee and Slovenian Adolescents. *Crisis:The Journal of Crisis Intervention and Suicide Prevention.* 2002; 23(3):127-132. https://doi.org/10.1027/0227-5910.23.3.127

Smith, M., & Alvarez, F. Identifying mortality factors fromMachine Learning using Shapley values–A case of COVID19. *Expert Systems with Applications*. 2021; 176: 114832.

Somasundaram, D. Collective trauma in northern Sri Lanka: a qualitative psychosocial-ecological study. *International Journal of Mental Health Systems*. 2007; 1(5), https://doi.org/10.1186/1752-4458-1-5

Somasundaram D & Jamunanantha C. Psychosocial consequences of war. In J. de Jong (Ed.), *Trauma, War, and Violence: Public Mental Health in Socio-cultural Context*. New York: Kluwer Academic/Plenum Publishers. 2002; 205-258.

Steel, Z, Chey, T, Silove, D, Mamane, C, Bryant, RA, Van Ommeren, M. Association of torture and other potentially traumatic events with mental health outcomes among populations exposed to mass conflict and displacement: a systemic review and meta-analysis. *Journal of the American Medical Association.* 2009; 302:537–549. DOI: 10.1001/jama.2009.1132

Stroufe,A. Psychopathology as an outcome of development. Development and Psychopathology. 1997; 9(2), 251 - 268. DOI: https://doi.org/10.1017/S0954579497002046

Štrumbelj, E.,&Kononenko, I. An efficient explanation of individual classifications using game theory. *The Journal ofMachine Learning Research*. 2010; 11:1–18.

Timshel, I., Montgomery, E.,& Dalgaard, N. A systematic review of risk and protective factors associated with family related violence in refugee families, *Child Abuse & Neglect*. 2017; 70:315-330. https://doi.org/10.1016/j.chiabu.2017.06.023.

Turner, S. What Is a Refugee Camp? Explorations of the Limits and Effects of the Camp, *Journal of Refugee Studies, Volume.* 2016; 29(2):139– 148, ttps://doi.org/10.1093/jrs/fev024

United Nations High Commissioner of Refugees. Projected Global Resettlement Needs. 2022. Available from: https://www.unhcr.org/en-us/protection/resettlement/60d320a64/projected-global-resettlement-needs-2022-pdf.html

United Nations High Commissioner of Refugees. Global Appeal. 2022. Available from: https://reporting.unhcr.org/globalappeal2022?page=6#_ga=2.195207212.124505709.1667315518-1562261994.1667315518

United Nations High Commissioner of Refugees. Refugee statistics. 2021. Available from: https://www.unrefugees.org/refugee-facts/statistics/

United Nations International Children's Emergency Fund. Nearly 50 million children "uprooted"worldwide.2022. Available from: https://www.unicef.org/press-releases/nearly-50-million-children-uprooted-worldwide

Valatheeswaran, C & Rajan, I. Sri Lankan Tamil Refugees in India: Rehabilitation Mechanisms, Livelihood Strategies, and Lasting Solutions**.***Refugee Survey Quarterly*. 2011; 30(2): 24-44. https://doi.org/10.1093/rsq/hdr005

Wang, D., Thunéll, S., Lindberg, U., Jiang, L., Trygg, J., & Tysklind, M. Towards better process management in wastewater treatment plants: Process analytics based on SHAP values for tree-based machine learning methods. *Journal of Environmental Management.* 2022; 301:113941. https://doi.org/10.1016/j.jenvman.2021.113941

Weathers, F., Litz, B., Keane, T., Herman, D., Steinberg, H., Huska, J., & Kraemer, H. The utility of the SCL-90-R for the diagnosis of war-zone related posttraumatic stress disorder. *Journal of Traumatic Stress*. 1996; 9 (1): 111-128. DOI: 10.1007/BF02116837.

Wikle, C.K., Datta, A., Hari, B.V., Boone, E.L., Sahoo, I., Kavila, I., Castruccio, S., Simmons, S.J., Burr, W.S. and Chang, W. An illustration of model agnostic explainability methods applied to environmental data. *Environmetrics*. 2022; 34(1): October. DOI: https://doi.org/10.1002/env.2772.

Williams, T & Williams,J. Open Ocean Bird Migratio. *IEE Proceedings F: Radar And Signal Processing*.1990; 13(2): 133-137. DOI: 10.1049/ip-f-2.1990.0019

Yang Liu & Allan Just. SHAPforxgboost: SHAP Plots for 'XGBoost". 2020. Available from: *https://github.com/liuyanguu/SHAPforxgboost*.

S1 Appendix

XGBoost Method

SHapley Additive exPlanations (SHAP) Method

 Click here to access/download;Figure;Figure 1.doc

Figure 1: SHAP values (impact on model output)

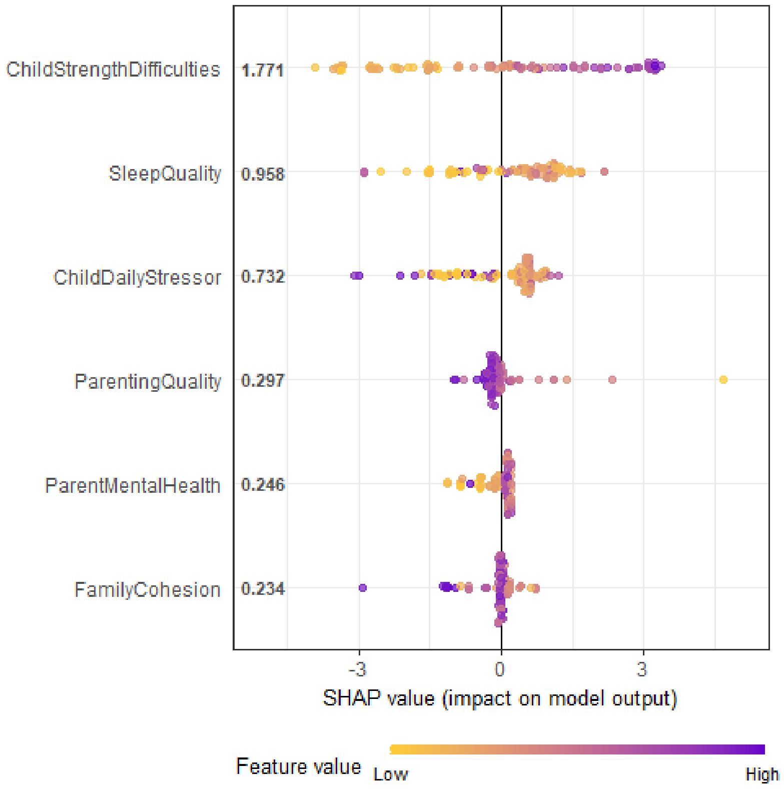

Click here to access/download
Supporting Information
Supplementary Material.doc
